# Response of soil microecology to different cropping practice under *Bupleurum chinense* cultivation

**DOI:** 10.1186/s12866-022-02638-3

**Published:** 2022-09-22

**Authors:** Li Liu, Hailu Cao, Yannan Geng, Quanfang Zhang, Xun Bu, Demin Gao

**Affiliations:** 1 School of Pharmacy, Shandong University of Traditional Chinese Medicine (TCM), Jinan, 250355 China; 2 Hengde Bencao (Beijing) Agricultural Technology Co., LTD, Beijing, 250100 China; 3 Department of pharmacy, Affiliated Hospital of Shandong University of TCM, Jinan, 250355 China; 4 Institute of Crop Germplasm Resources, Shandong Academy of Agricultural Sciences, Jinan, 250100 China

**Keywords:** *Bupleurum chinense*, Cropping practice, Soil microecology

## Abstract

The effects of cropping practices on the rhizosphere soil physical properties and microbial communities of *Bupleurum chinense* have not been studied in detail. The chemical properties and the microbiome of rhizosphere soil of *B. chinense* were assessed in the field trial with three cropping practices (continuous monocropping, *Bupleurum*-corn intercropping and *Bupleurum*-corn rotation). The results showed cropping practices changed the chemical properties of the rhizosphere soil and composition, structure and diversity of the rhizosphere microbial communities. Continuous monocropping of *B. chinense* not only decreased soil pH and the contents of NO_3_^−^-N and available K, but also decreased the alpha diversity of bacteria and beneficial microorganisms. However, *Bupleurum*-corn rotation improved soil chemical properties and reduced the abundance of harmful microorganisms. Soil chemical properties, especially the contents of NH_4_^+^-N, soil organic matter (SOM) and available K, were the key factors affecting the structure and composition of microbial communities in the rhizosphere soil. These findings could provide a new basis for overcoming problems associated with continuous cropping and promote development of *B. chinense* planting industry by improving soil microbial communities.

**Supplementary Information:**

The online version contains supplementary material available at 10.1186/s12866-022-02638-3.

## Introduction

*Bupleurum chinense* (Apiaceae) is an important medicinal plant that has been used in China and other Asian countries for thousands of years. The plant has many important properties, such as anti-inflammatory [1], liver-protecting [2], anti-depressant [3], anti-tumor [4], and immunomodulatory [5] activities, and is widely used in the clinical treatment of fever, influenza, malaria, distending pain in the chest, menstrual disorders, and other symptoms. The active components of Bupleuri radix mainly include saponins [6], polysaccharides [7], essential oil [8], flavones [9], and coumarin [10]. These compounds are not only related to *Bupleurum* germplasm, but are also influenced by the production environment and cropping practices [9, 11].

In the recent decades, as a result of rapid development and large-scale cultivation, the planting area of *B. chinense* has expanded substantially. However, due to the limited available land and maximum economic benefits, the continuous cultivation of *B. chinense* is becoming increasingly popular. Studies showed that long-term continuous cropping resulted in decreased abundance of beneficial microorganisms in soil, the increase in pathogenic microorganisms, and the decrease in yield and quality of medicinal materials [12]. For example, the continuous planting of American ginseng [13] and *Sophora flavescens* [14] not only decreased weakened soil microbial diversity and amassed fungal root pathogens, but also changed soil physical properties, resulting in decreased crop yield and quality.

Studies have shown that multiple cropping system [characterized by more than one crop grown together, either mixed in space (intercropping) or time (crop rotation)] can effectively alleviate the problems associated with monocropping. Intercropping, in which two or more crops are planted in the same field, can increase the absorption of trace elements, improve soil fertility [15] and reduce the risk of pests and diseases [16]. For example, the intercropping of turmeric, ginger and patchouli not only changed the soil physical properties and the microbial community structure, but also improved the quality of patchouli [17].

Crop rotation involves the systematic rotation of different types of crops in the same field. Crop rotation can balance soil nutrients, improve soil chemical properties, increase the abundance of beneficial microorganisms, and enhance disease resistance [18]. For example, the rotation *Pinellia ternata-*wheat improved soil microecological environment, enriched beneficial microorganisms and diminished pathogenic microorganisms [19].

However, the effects of cropping practices on the rhizosphere soil microecology of *B. chinense* have not been studied in detail, especially the dynamic changes in rhizosphere soil microorganisms and soil physical and chemical properties after continuous planting of *B. chinense*. This lack of knowledge affects the development of *B. chinense* planting industry.

The objective of the study was to investigate the effect of cropping practices on soil rhizosphere microecology of *B. chinense.* A high-throughput Illumina MiSeq sequencing platform was used to determine the microbial community structures in the *B. chinense* rhizosphere soil in different cropping practices. The chemical properties of rhizosphere soil were determined by the previously reported methods [20]. Our study could provide a new basis for overcoming continuous-cropping obstacles and promote development of *B. chinense* planting industry.

## Materials and methods

### Field experiment

The experimental site was a trial plot of Shandong University of Chinese Medicine, Shandong Province, China (117°22′54″ E 36°35′27″ N, altitude 524 m). The annual average sunshine was 2647.6 h, and the sunshine rate was 60%. The annual average temperature was 12.8 °C, and the annual average precipitation was 600.8 mm. The soil type was brown soil.

The field experiment was conducted from June 2016 to October 2020. The field trial area was divided into three plots of 5 × 5 square meters each. Three treatments were implemented: *B. chinense* continuous cultivation (BCC), *B. chinense* intercropped with corn (BIC) and growing corn after *B. chinense* (BCR); each treatment had three repetitions. Cultivation time and sowing of *Bupleurum* seeds and corn are shown in Table 1. All the experimental plots were subjected to the same field management practices, including manual weeding, no fertilizer and no watering. During the experiment, the soil microbial and chemical characteristics were analyzed for three consecutive years to assess temporal variation. After the flowering of *B. chinense* in September of the second year, soil samples of the three cropping treatments were taken for comparative analysis.

**Table 1:** Stages of field experiment featuring *Bupleurum chinense* cultivation patterns

	2016.6. 15	2017	2018.10.18	2019.4.17	2019.6.15	2020.9.25
Plot I	*B. chinense*	*B. chinense*	Harvesting	–	*B.chinense*	Soil sampling analysis (BCC)
Plot II	*B. chinense*	*B.chinense*	Harvesting	Planting corn	*B. chinense*	Soil sampling analysis (BIC)
Plot III	*B.chinense*	*B.chinense*	Harvesting	Planting corn	Corn	Soil sampling analysis (BCR)

-, no crops planted

*BCC Bupleurum chinense* continuous cultivation, *BIC B. chinense* intercropped with corn, *BCR* growing corn after *B. chinense*

### Collection of soil samples

Rhizosphere soil samples were collected in October 2020. Rhizosphere soil samples from 30 plant were collected from five different sites using the Z-type method in each experimental plot. Then, 30 rhizosphere samples were combined into a composite sample [21]. There were triplicate rhizosphere soil samples for each treatment. Firstly, the loose soil was shaken off from roots (the depth of roots was about 10 cm), and the soil closely adhering to the root system was sampled as rhizosphere soil by brushing it off [22]. The collected soil was then placed in a sealed sterile bag and taken back to the laboratory. Each soil sample was divided into two subsamples: one for chemical analysis, and the other was stored at − 20 °C for microbial analysis.

### Chemical properties

After air-drying, the pH value of the soil was measured using a pH meter (pHS-3S) (2.5:1 water:soil ratio), and the contents of soil organic matter (SOM) (SOM = SOC × 1.724), available phosphorus (Ava-P), and available potassium (Ava-K) were determined by the methods reported by Qu et al. [23]. Determination of NO_3_^−^-N and NH_4_^+^-N in soil was done by UV spectrometry as reported by Xing et al. [24].

### DNA extraction

A PowerSoil DNA Isolation Kit (MoBio Laboratories, Carlsbad, CA, USA) was used to extract DNA from soil samples. Each soil sample was extracted according to the PowerSoil kit manufacture’s protocol. The extracted DNA was eluted using 100 μL sterile water, quantified using a NanoDrop 2000 spectrophotometer (Thermo Scientific, Canada) and stored at − 20 °C for further use.

### PCR amplification and Illumina MiSeq sequencing

The V4-V5 region of bacterial 16S rDNA was amplified using the primers 515F 5′-GTGCCAGCMGCCGCGGTAA-3′ and 926R 5′-CCGTCAATTCMTTTGAGTTT-3′, whereas the fungal ITS1 region was amplified using F 5′-CTTGGTCATTTAGAGGAAGTAA-3′, and R 5′-GCTGCGTTCTTCATCGATGC-3′). The primers also contained the Illumina 5′-overhang adapter sequences for two-step amplicon library building, following manufacturer’s instructions. The initial PCR reactions were carried out in 50 μL reaction volumes with 1–2 μL DNA template, 200 μM dNTPs, 0.2 μM of each primer, 5 × reaction buffer 10 μL, and 1 U Phusion DNA Polymerase (New England Biolabs, USA). PCR conditions consisted of initial denaturation at 94 °C for 2 min, followed by 25 cycles of denaturation at 94 °C for 30 s, annealing at 56 C for 30 s and extension at 72 °C for 30 s, with a final extension at 72 °C for 5 min [21]. The barcoded PCR products were purified using a DNA gel extraction kit (Axygen, USA) and quantified using an FTC − 3000 TM real-time PCR (Funglyn Shanghai). The PCR products from different samples were mixed at equal ratios. The second step PCR with dual 8 bp barcodes was used for multiplexing. Eight-cycle PCR reactions were used to incorporate two unique barcodes to either end of the amplicons. Cycling conditions consisted of 1 cycle at 94 °C for 3 min, followed by 8 cycles at 94 °C for 30 s, 56 °C for 30 s and 72 °C for 30 s, and a final extension cycle at 72 °C for 5 min. The library was purified using a DNA gel extraction kit (Axygen, USA) and sequenced by 2 × 250 bp paired-end sequencing on a Novaseq platform using a Novaseq 6000 SP 500 Cycle Reagent Kit (Illumina USA) at TinyGen Bio-Tech (Shanghai) Co., Ltd.

### Illumina data analysis

The raw fastq files were demultiplexed based on the barcode. The PE reads for all samples were run through Trimmomatic (version 0.35) to remove low quality base pairs using the parameters SLIDINGWINDOW 50:20 and MINLEN 50. The trimmed reads were then cut to separate adaptors using Cutadapt (version 1.16) and were merged using FLASH program (version 1.2.11) with default parameters.

The sequences were analyzed using a combination of software Mothur (version 1.33.3), UPARSE (usearch version v8.1.1756, http://drive5.com/uparse/), and R (version 3.6.3). The demultiplexed reads were clustered at 97% sequence identity into operational taxonomic units (OTUs). The singleton OTUs were deleted using the UPARSE pipeline (http://drive5.com/usearch/manual/uparse cmds.html). The representative OTU sequences of bacteria were assigned taxonomically against the Silva 128 database (ITS in Unite database) with confidence score ≥ 0.6 by the classify.seqs command in Mothur.

The indices of alpha diversity were calculated by Mothur. For the beta diversity analysis, the Weighted UniFrac distance algorithm was used to calculate the distance between samples. In LEfSe analysis, the linear discriminant analysis (LDA) score was computed for taxa differentially abundant between the two treatments. A taxon at *P* < 0.05 (Kruskal–Wallis test) and log10[LDA] ≥ 2.0 (or ≤ − 2.0) was considered significant. Statistical and visual analysis of dilution curves, community structure histogram, NMDS and RDA were performed using R language (Version 3.6.3). PICRUSt software and FUNGuild software were used to predict the function of bacterial and fungal gene sequences, respectively.

All statistical analyses were performed using SPSS Statistics 21.0. The data on the chemical properties and microbial diversity of rhizosphere soil were analyzed by Duncan’s multiple range test. Differences in the relative abundances of microbial taxa among treatments were analyzed using one-way analysis of variance (ANOVA) at the 0.05 probability level.

## Results

### The effect of cropping practices on the rhizosphere soil chemical properties

The chemical properties of *B. chinense* rhizosphere soil in different treatments are shown in Table 2. Compared with intercropping and crop rotation, soil pH and the contents of NO_3_^−^-N and Ava-K decreased after continuous planting of *B. chinense*, but the Ava-P content increased. The chemical parameters of rhizosphere soil differed significantly among the treatments*,* except for the NO_3_^−^-N content.

**Table 2:** Chemical properties of rhizosphere soil of *Bupleurum chinense* from different cropping practices

Samples	pH	SOM(g/kg)	NO_3_^−^-N (mg/kg)	NH_4_^+^-N (mg/kg)	Ava-K (mg/kg)	Ava-P (mg/kg)
BCC	6.67 ± 0.04^a^	18.32 ± 0.18^a^	49.53 ± 2.23^a^	43.58 ± 0.49^a^	154.12 ± 11.87^a^	92.19 ± 3.30^a^
BIC	7.94 ± 0.07^b^	17.39 ± 0.32^b^	93.98 ± 4.15^b^	38.97 ± 0.78^b^	186.54 ± 4.01^b^	60.92 ± 1.82^b^
BCR	8.07 ± 0.07^c^	19.07 ± 0.30^c^	95.55 ± 2.28^b^	62.27 ± 1.89^c^	220.19 ± 12.88^c^	73.28 ± 1.44^c^

Different lowercase letters indicate significant differences among different samples (*P* < 0.05; Duncan’s multiple range test). SOM, soil organic matter. Ava-K, available potassium. Ava-P, available phosphorus

### Amplicon sequencing and rarefaction curves

To characterize the microbiome in the *B. chinense* rhizosphere soil in different cropping practices, nine samples were sequenced by Illumina MiSeq. The amplicon sequencing resulted in 450,038 effective reads of bacterial 16S rRNA genes and 437,141 effective reads of fungal ITS region. Based on 97% similarity, the OTUs of microbial community in the rhizosphere soil were obtained. The results are shown in supplementary Table 1.

To construct rarefaction curves, the dataset was flattened according to the minimum number of sample sequences. The rarefaction curves of nine rhizosphere soil samples were constructed based on the number of OTUs observed (Supplementary Fig. 1). The rarefaction curves showed that the number of OTUs rose sharply and then gradually flattened out, indicating that the sequencing library reached saturation. Therefore, it could be used for analyzing the diversity of microorganisms in the rhizosphere soil of *B. chinense*.

### Alpha diversity of bacterial and fungal communities

The alpha diversity represents the measurement of within-community microbial diversity (Table 3). Theoretically, the larger the Shannon index or the smaller the Simpson index, the higher the community diversity. According to the Shannon index, the bacterial richness was highest (6.513) in the rhizosphere soil of the rotation of *B. chinense* and corn, followed by continuous monocropping (6.421) and intercropping of *B. chinense* and corn (6.328). The Simpson index analysis confirmed the above-mentioned diversity analysis. Shannon index and Simpson index values for fungal communities in the rhizosphere of *B. chinense*-corn intercropping were 4.401 and 0.029, respectively, followed by those of rotation with corn (4.250 and 0.033, respectively), and the lowest diversity values were in *B. chinense* monocropping (4.201 and 0.049, respectively). The results showed that the rotation and intercropping of *B. chinense* with corn were the main factors affecting the diversity of, respectively, bacteria and fungi in the rhizosphere. In summary, the cropping practices had an important effect on the diversity of rhizosphere microorganisms.

**Table 3:** Shannon and Simpson indices of rhizosphere microbial community of *Bupleurum chinense* under different cropping practices

Sample	Bacteria		Fungi	
	Shannon	Simpson	Shannon	Simpson
BCC	6.421 ± 0.019^a^	0.006 ± 0.001a	4.201 ± 0.218a	0.049 ± 0.020a
BIC	6.328 ± 0.046^ab^	0.006 ± 0.000a	4.401 ± 0.197a	0.029 ± 0.005a
BCR	6.513 ± 0.074^ac^	0.004 ± 0.000b	4.250 ± 0.192a	0.033 ± 0.008a

Shannon and Simpson indices represents the diversity of bacteria or fungi. Values are expressed as mean ± SD (*n* = 3). Different lowercase letters indicate significant differences among different samples (*P* < 0.05; Duncan’s multiple range test)

### Beta diversity of bacterial and fungal communities

**Fig. 1:**
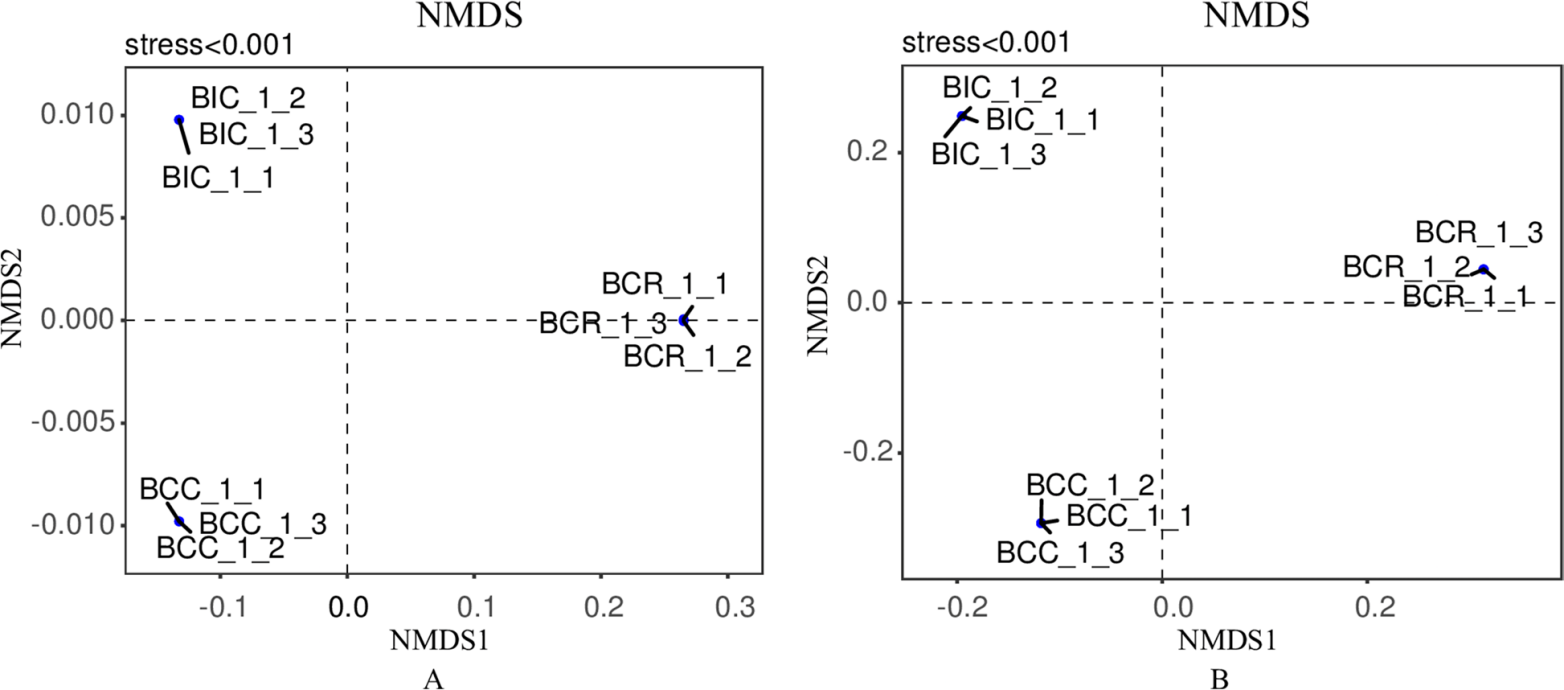
The non-metric multidimensional scale (NMDS) map of the unweighted UniFrac distance representings the microbial community structure. **A** Bacteria, **B** Fungi

In order to shed more light on the differences in microbial community structure, NMDS analysis was performed based on the Weighted UniFrac distance (Fig. 1), and the samples could be divided into three groups, according to the species composition in the *B. chinense* rhizosphere. There were similarities in the structure of microbial communities within the treatments and significant differences in the structure among the treatments, which indicated that the cropping practices in the same field strongly influenced the composition of microbial communities in the *B. chinense* rhizosphere.

### The composition and structure of the bacterial community

In order to clarify the microbial community structure in the *B. chinense* rhizosphere, two taxonomic levels (phylum and genus) were analyzed.

**Fig. 2:**
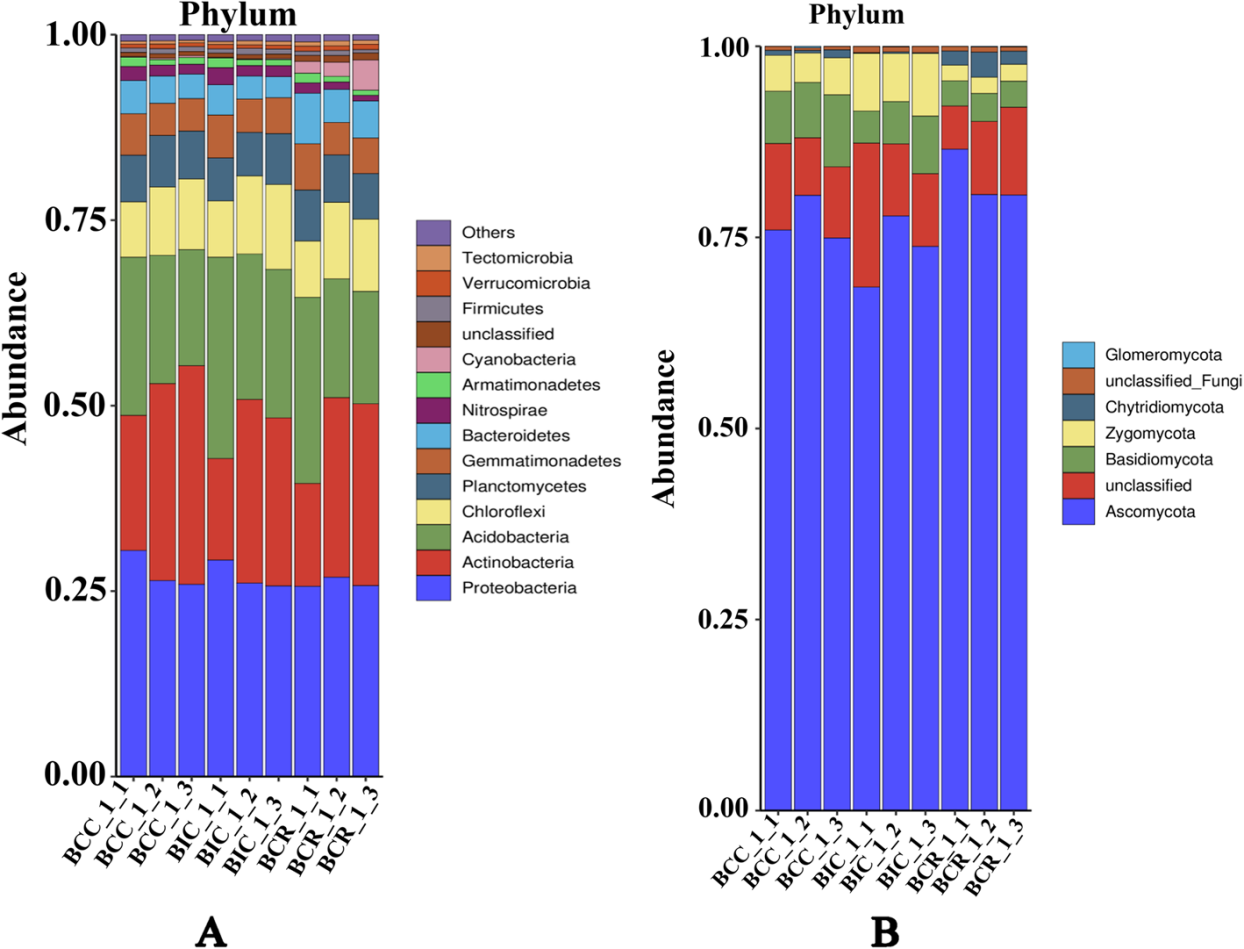
Relative abundance of microbial phyla under various cropping practices. **A** Bacteria, **B** Fungi. BCC_1_1, BCC_1_2 and BCC_1_3 represents three repeated samples of rhizosphere soil collected in October 2020. BIC and BCR are also applicable

As shown in Fig. 2A, 13 bacterial phyla were detected in the soil from different cropping practices. The dominant bacterial phyla in the *B. chinense* rhizosphere soil were Proteobacteria, followed by Actinobacteria, Acidobacteria, and Chloroflexi. As compared to BIC and BCC, continuous cropping of *B. chinense* for 3 years resulted in higher abundance of Proteobacteria and Actinobacteria, but in lower abundance of Acidobacteria.

**Fig. 3:**
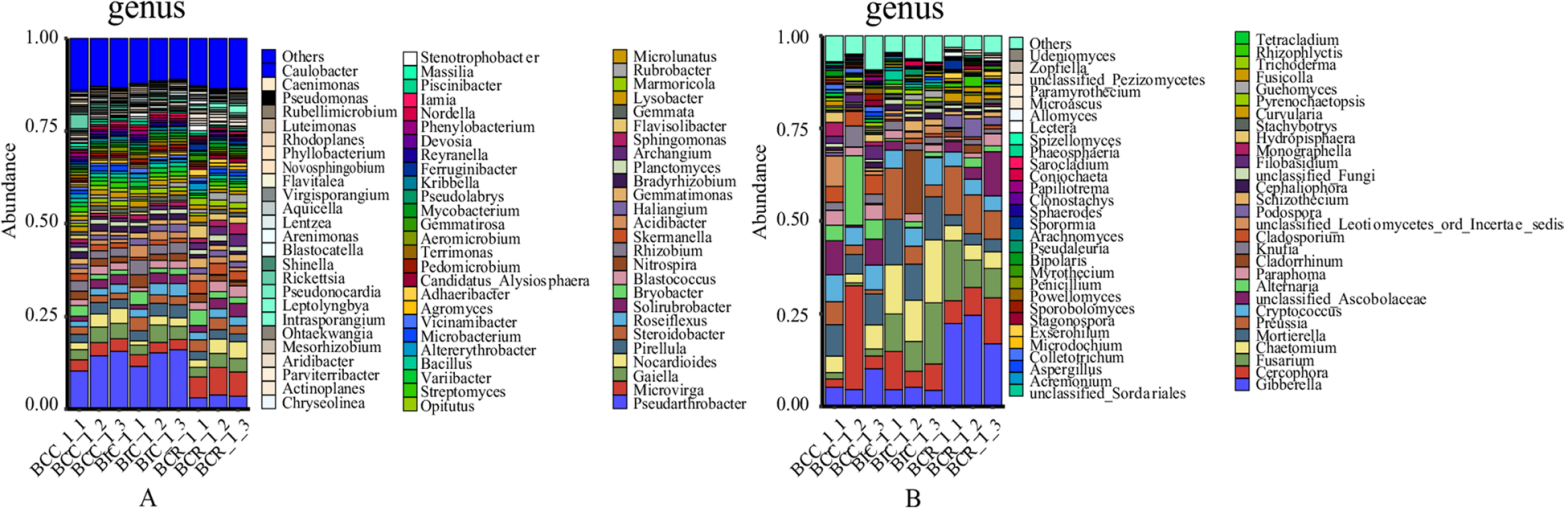
Relative abundances of microbial genera with significant differences under various cropping practices. **A** Bacteria, **B** Fungi

**Fig. 4:**
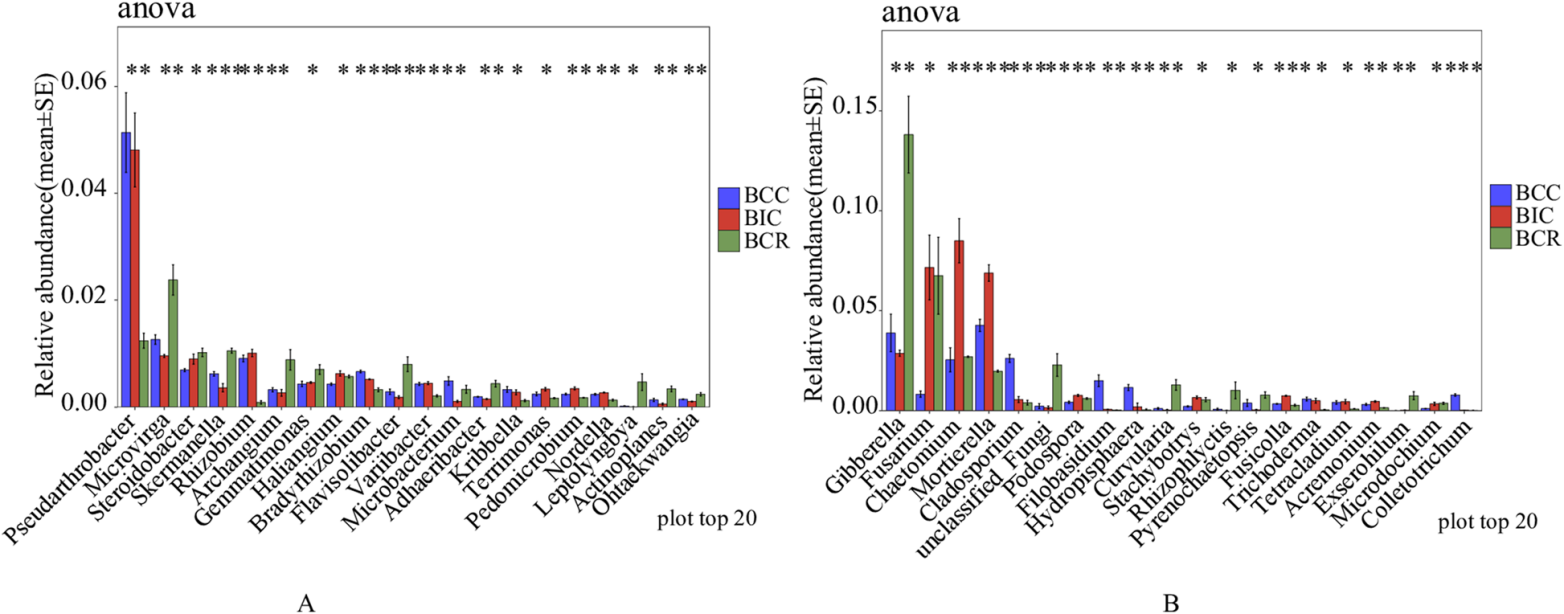
Relative abundances of the top 20 bacterial and fungal genera in the rhizosphere of *Bupleurum chinense* in different cropping practices. Vertical bars represent the mean standard deviation of three replicates. Asterisks with different letters indicate significant differences (*P* < 0.05) based on LSD. **A** Bacteria, **B** Fungi

At the genus level (Fig. 3A), 79 bacterial genera were detected in the rhizosphere soil from different cropping practices. The dominant genera were *Pseudarthrobacter*, *Microvirga*, *Gaiella*, *Nitrospira*, and *Pirellula*. Compared with the intercropping and rotation of *B. chinense* and corn, the relative abundance of *Pseudarthrobacter* and *Gaiella* increased after continuous cropping. However, the relative abundance of *Microvirga* and *Nitrospira* showed a downward trend after continuous cropping and intercropping (Fig. 4A).

### The composition and structure of the fungal community

As shown in Fig. 2B, five fungal phyla were detected in the soil from different cropping practices. The dominant fungal phyla were Ascomycota, Basidiomycota and Zygomycota. The relative abundance of Ascomycota decreased after continuous cropping and intercropping, but increased after rotation with corn. The relative abundance of Basidiomycetes increased after continuous cropping, but decreased after intercropping and rotation.

At the genus level (Fig. 3B), 60 fungal genera were detected in the soil from different cropping practices. The dominant fungal genera in the rhizosphere soil were *Gibberella*, *Cercophora*, *Fusarium*, *Chaetomium*, *Mortierella*, *Preussia*, *Cryptococcus*, *Alternaria*, *unclassified*_*Ascobolaceae*, *Cladorhinum*, *Paraphoma*, *Knufia*, and *Cladosporium*. After 3 years of continuous cultivation of *B. chinense*, the relative abundance of *Cercophora*, *Cryptococcus*, *Alternaria*, *Paraphoma* and *Cladosporium* increased, but the relative abundance of *Chaetomium*, *Mortierella*, *Preussia* and *Cladorrhinum* significantly decreased (Fig. 4B).

### Correlation analysis of dominant microorganisms and soil properties

**Fig. 5:**
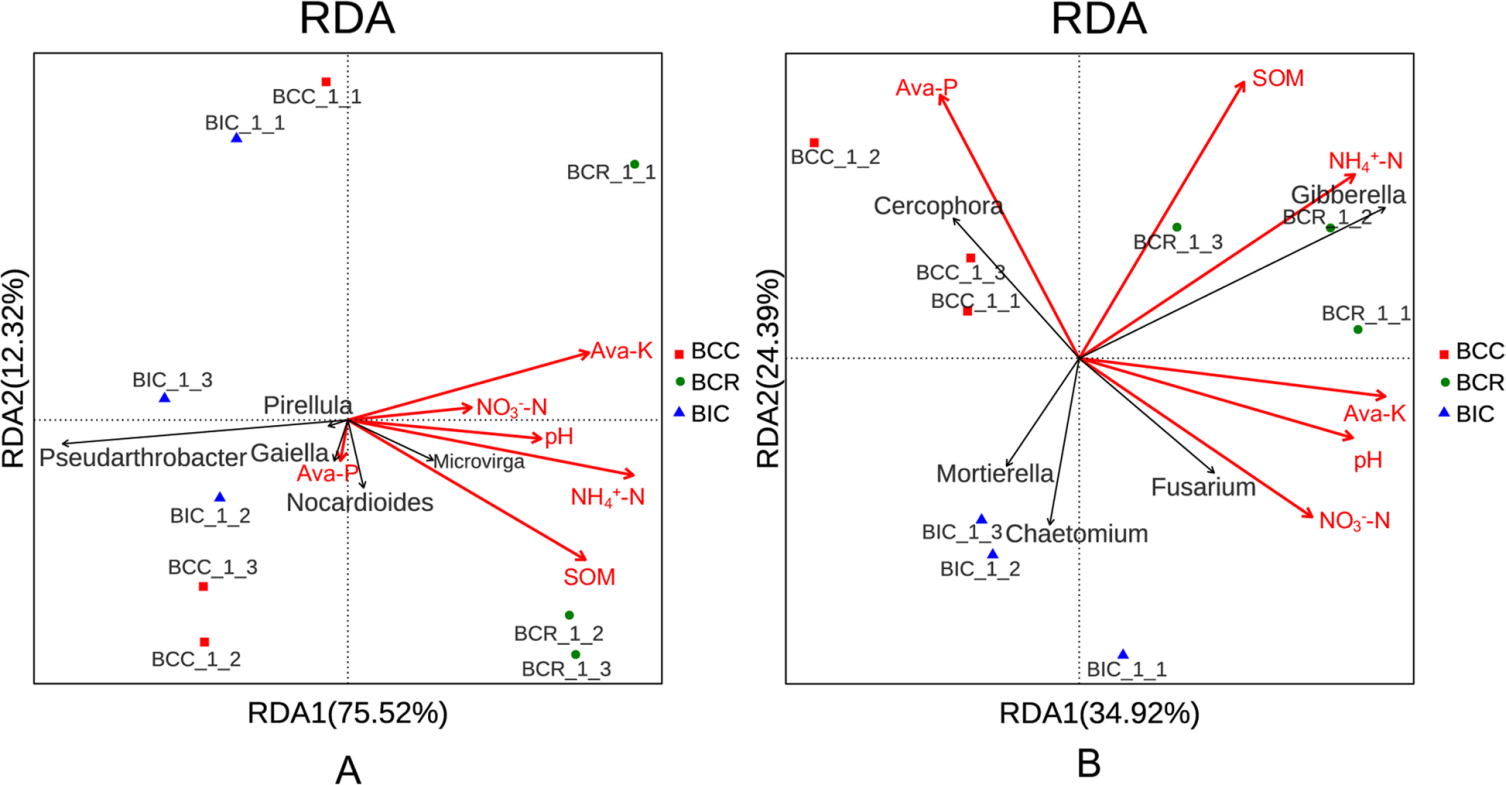
Redundancy analysis (RDA) on dominant fungal genera (**A**) and dominant bacterial genera (**B**) constrained by soil chemical properties

Soil chemical properties were important explanatory factors that determined the clustering patterns of soil microbial communities in different cropping treatments [25]. The chemical properties of the *B. chinense* rhizosphere soil were significantly different under different cropping practices (Table 2). Therefore, redundancy analysis (RDA) was conducted on the relative abundance of dominant bacterial and fungal genera and soil chemical factors (Fig. 5). The results showed that the cumulative variation explained by the soil chemical properties was 87.84 and 59.31% for bacteria and fungi, respectively, indicating that explanatory variables had a significant influence on the structure of microbial communities. The effects of soil chemical properties on bacteria and fungi were in the order of NH_4_^+^-N > SOM > Ava-K > pH > NO_3_^−^-N > Ava-P and NH_4_^+^-N > SOM > Ava-K > Ava-P > pH > NO_3_^−^-N, respectively (Fig. 5). In conclusion, NH_4_^+^-N, SOM and Ava-K were the main chemical properties that affected the microbial abundance and composition in the *Bupleurum* rhizosphere soil.

### Biomarker analysis

**Fig. 6:**
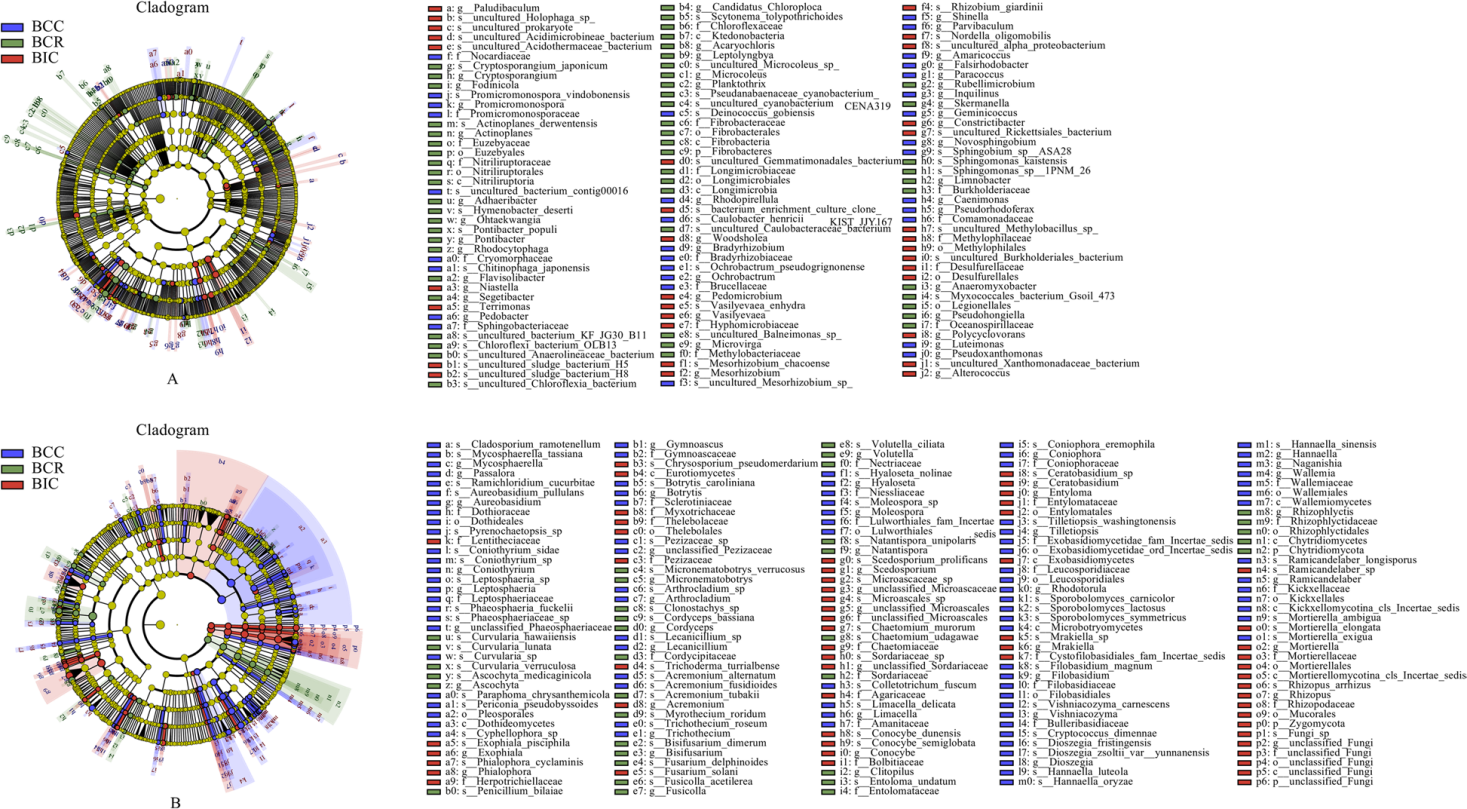
LEfSe analysis of microbial community differences in the rhizosphere of *Bupleurum chinense* in different cropping practices. **A** Bacteria, **B** Fungi

In order to identify the dominant microbial biomarkers in the *B. chinense* rhizosphere soil under different cropping practices, the linear discriminant analysis (LDA) effect size (LEfSe) was carried out (Fig. 6). The LDA results identified 30, 33 and 55 bacterial biomarkers in continuous monocropping, intercropping and rotation with corn*,* respectively (Fig. 6A). The most abundant bacterial family was Comamonadaceae from *B. chinense* continuous monocropping soil. *Rhizobium giardinii*, Desulfurellaceae and Burkholderiales were abundant in the rhizosphere of *B. chinense* intercropped with corn, whereas Methylobacteriaceae and Microvirga were significantly enriched in the rhizosphere of *B. chinense* in rotation with corn.

For the fungal community, we identified 92, 57 and 34 fungal biomarkers in continuous monocropping, intercropping and rotation with corn*,* respectively (Fig. 6B). The relatively abundant biomarker fungal taxa included Dothideomycetes and Pleosporales in the *B. chinense* continuous monocropping, Chaetomiaceae, Mortierellaceae and Zygomycota in *B. chinense* intercropped with corn, and Nectriaceae, Chytridiomycetes and Rhizophlyctidales in *B. chinense* in rotation with corn.

### Functional analysis

**Fig. 7:**
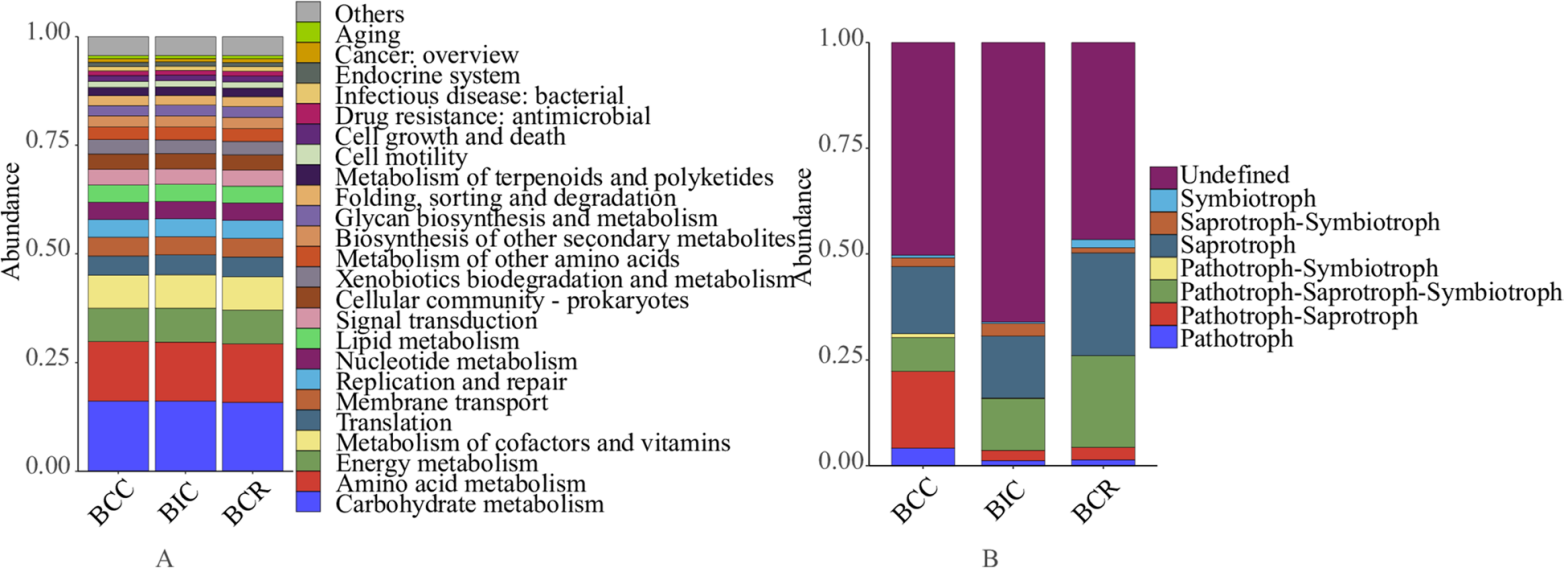
Prediction of microbial community function in the rhizosphere of *Bupleurum chinense* in different cropping practices. **A** Bacteria, **B** Fungi

In order to explore the functional changes in soil bacteria in different cropping treatments, six categories of biological metabolic pathways (main functional levels) were identified by comparing with KEGG database. It included metabolism, genetic information processing, environmental information processing, cellular processes, human diseases, and organismal systems, accounting for 66, 11, 8, 7, 5, and 3%, respectively. In addition, 24 sub-functions such as amino acid metabolism, energy metabolism, metabolism of cofactors and vitamins, and translation were found by analyzing the secondary functional layers of predictive genes (Fig. 7A).

The secondary pathways of *B. chinense* under different cropping treatments were similar, that is, carbohydrate metabolism and amino acid metabolism were significantly higher than the other metabolic pathways. The carbohydrate metabolism and amino acid metabolism were higher in continuous cropping of *B. chinense* than in other cropping patterns.

According to the FUNGuild database, at least eight nutrient patterns were detected in this study, whereby saprophytes were most abundant, followed by pathotroph-saprotroph-symbiotroph and pathotroph-saprotroph patterns. The relative abundance of fungal functions varied significantly among different treatments. Compared with intercropping and rotation, pathotrophs, pathotroph-symbiotrophs, and pathotroph-saprotrophs were most abundant in continuous cropping (Fig. 7B).

## Discussion

Soil chemical characteristics are the important indices for evaluating soil quality. Cropping practices influenced not only the chemical properties of soil, but also governed the composition of rhizosphere microorganisms [26]. Therefore, elucidating the changes in soil chemical properties can provide a basis for characterizing soil productivity under different cropping practices. A decrease in soil nutrients was associated with a decrease in diversity of rhizosphere microbial community, which is one of the main causes of problems with crop continuous cropping. However, intercropping and rotation increased soil nutrient contents, thereby increasing the diversity of rhizosphere microbial community and alleviating continuous cropping problems [27].

In our study, the contents of pH, NO_3_^−^-N and Ava-K decreased after continuous cropping of *B. chinense,* but increased after intercropping and rotation with corn. Studies have shown that continuous monocropping systems have a negative impact on soil function and sustainability [28, 29]. Soil nutrient contents such as SOM, Ava-P, Ava-K, NO_3_^−^-N and NH_4_^+^-N showed a decreasing trend after continuous cropping. However, rotation or intercropping with corn effectively alleviated this decline and imbalance in soil nutrients caused by continuous monocropping [26, 30]. Our experiment, confirming the above findings, contributed to the sustainable development of *B. chinense* planting industry through rotation of *B. chinense* with corn. Studies have shown that soil organic matter is a key factor affecting soil microbial community diversity, and high soil organic matter content is conducive to improving soil bacterial community diversity [31]. In our study, the rhizosphere soil bacterial diversity increased after *B. chinense* rotation, but decreased after *B. chinense* intercropping with corn, which might have been related to the decrease in organic matter content after *B. chinense* intercropping and the increase of organic matter after rotation.

Higher soil microbial community diversity is indicative of higher soil health and plant productivity [32]. Soil microbial diversity not only has an important impact on soil quality, function and sustainability [33], but also is a key factor in the control of pathogenic microorganisms [34]. Therefore, the loss of soil microbial diversity and function is one of the reasons for poor crop growth under continuous monocropping. In order to ensure the accuracy and reliability of the test results, the soil microbial and chemical properties were analyzed for three consecutive times during the experiment. Our results showed the cropping practice of *B. chinense* significantly affected the structure and composition of soil microbial community. In *B. chinense* continuous monocropping, alpha diversity decreased, but this change could be alleviated by rotation or intercropping with corn. Similar results were also obtained in continuous monocropping of sugar beet [22] and *Coptis chinensis* Franch [35]., intercropping of potato with onion and tomato [36] and intercropping of black pepper and vanilla [30], as well as rotation of Brassica vegetables with eggplant [26], and *Pinellia ternata* with wheat [19].

Beta diversity showed that cropping practices had a strong influence on the soil microbial community. In other words, the use of different cropping systems may lead to significant differences in the structure of microbial communities in soil [37]. Importantly, changes in microbial community structure and composition usually are associated with changes in plant metabolic capacity, biodegradation, disease inhibition, and other functions [38].

In our study, the continuous monocropping of *B. chinense* strongly reduced the abundance of beneficial microorganisms, such as *Microvirga*, *Haliangium*, *Chaetomium*, *Mortierella*, *Preussia*, and *Cladorrhinum*. These rhizosphere microorganisms played an important role in plant growth and the inhibition of pathogenic microorganisms [39–43]. By contrast, some potentially pathogenic microorganisms, such as *Cercophora* [44], *Alternaria*, *Paraphoma*, *Cladosporium* [45], *Monographella* [46], *Hydropisphaera* [47], and *Colletotrichum* were significantly amplified. For example, *Alternaria* and *Paraphoma* can cause root rot of *B. chinense* [25, 48], and *Colletotrichum* can infect leaves to produce disease spots [49]*.* In this study, ecological functions in the rhizosphere soil of *B. chinense* in different cultivation modes were predicted. Among fungi, compared with other groups, the abundance of pathotrophs, pathotroph-symbiotrophs, and pathotroph-saprotrophs, which may cause plant diseases, increased significantly in continuous cropping.

## Conclusion

The results showed that continuous cropping of B. chinense resulted in the decrease of pH, NO_3_^−^-N and Ava-K in the rhizosphere soil, and the decrease in rhizosphere bacterial and fungal α-diversity. The relative abundance of beneficial microorganisms was reduced, and intercropping and rotation could alleviate these problems. Soil chemical properties, especially the contents of NH_4_^+^-N, SOM and Ava-K, influenced the microbial structure and composition of the *B. chinense* rhizosphere soil. These findings could provide a new basis for overcoming the problems associated with continuous cropping and promoting development of *B. chinense* planting industry by improving soil microbial communities.

## Supplementary Information

**Additional file 1: Supplement Table 1**. High-throughput results for bacteria and fungi in the rhizosphere soil of *Bupleurum chinese* under different cropping practices.

**Additional file 2: Supplement Figure 1**. Rarefaction curves of *Bupleurum chinense* samples in different cropping practices. (A) Bacteria, (B) Fungi.

## Data Availability

All sequencing data were deposited into NCBI’s Sequence Read Archive (SRA) database with the Bioproject number PRJNA777373.
