# Supplementary material for: Response of soil microecology to different cropping practice under Bupleurum chinense cultivation
Source: BMC Microbiol. 2022 Sep 22;22:223. doi: 10.1186/s12866-022-02638-3 (PMC9494904; doi:10.1186/s12866-022-02638-3)
Supplement: Supplementary file 1 — Additional file 1: Supplement Table 1. High-throughput results for bacteria and fungi in the rhizosphere soil of Bupleurum chinese under different cropping practices. [file 12866_2022_2638_MOESM1_ESM.docx]

Supplement

Table 1 High-throughput results for bacteria and fungi in the rhizosphere soil of *Bupleurum chinese* under different cropping practices

| Sample | Bacteria | | Fungi | |
| --- | --- | --- | --- | --- |
|  | Reads | OTU | Reads | OTU |
| BCC_1_1 | 43354 | 2640 | 43785 | 644 |
| BCC_1_2 | 54378 | 2924 | 54404 | 686 |
| BCC_1_3 | 54643 | 2888 | 54784 | 743 |
| BIC_1_1 | 45319 | 2560 | 40985 | 462 |
| BIC_1_2 | 49355 | 2644 | 51261 | 561 |
| BIC_1_3 | 54286 | 2768 | 46795 | 556 |
| BCR_1_1 | 44732 | 2483 | 44969 | 488 |
| BCR_1_2 | 49196 | 2647 | 49327 | 519 |
| BCR_1_3 | 54775 | 2716 | 50831 | 561 |

Note: Reads represent the effective sequence of bacteria or fungi. OTU represents the classification operating unit of bacteria or fungi. BCC_1_1, BCC_1_2 and BCC_1_3 represents three repeated samples of rhizosphere soil collected in October 2020, BIC and BCR are also applicable.
