# Supplementary figures and images for: Response of soil microecology to different cropping practice under Bupleurum chinense cultivation
Source: BMC Microbiol. 2022 Sep 22;22:223. doi: 10.1186/s12866-022-02638-3 (PMC9494904; doi:10.1186/s12866-022-02638-3)

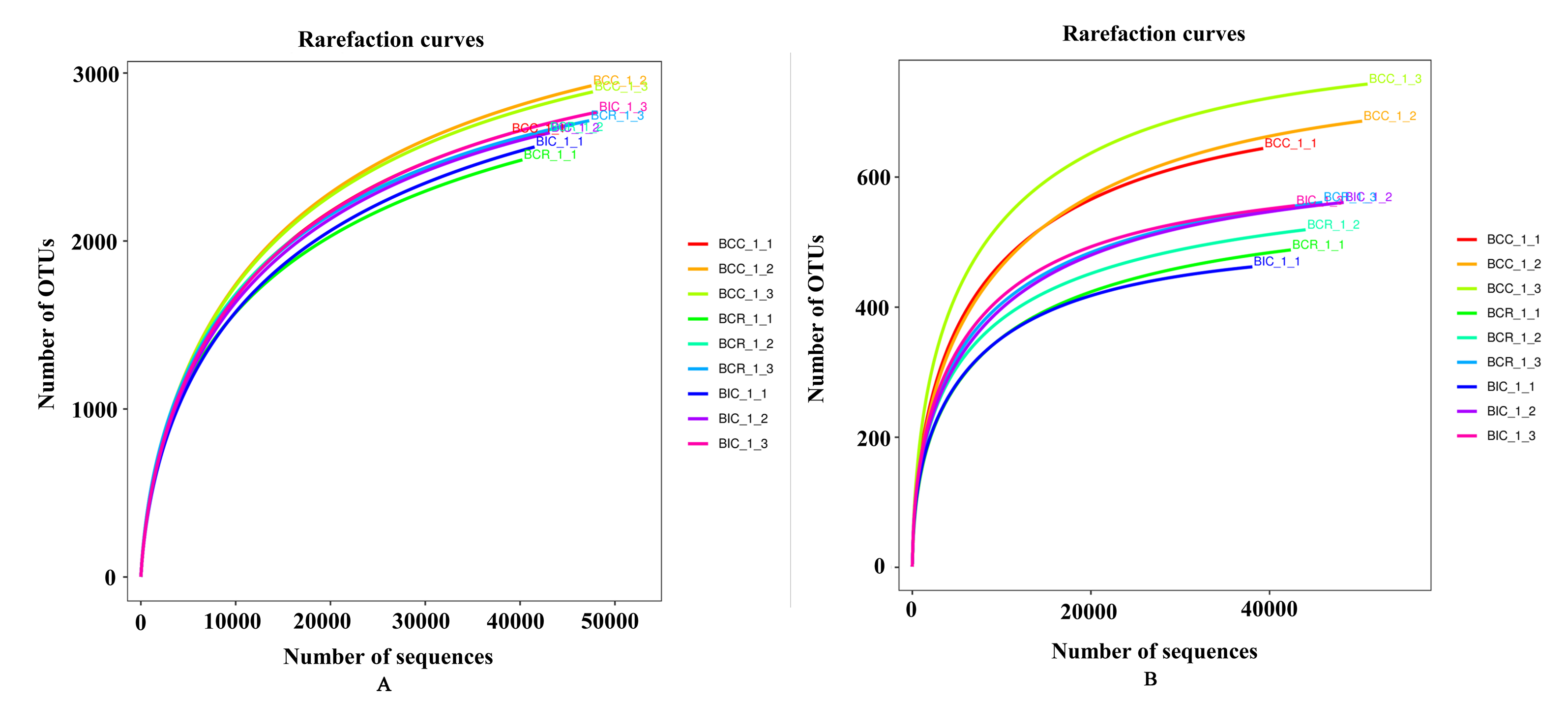

Supplement: Supplementary file 2 — Additional file 2: Supplement Figure 1. Rarefaction curves of Bupleurum chinense samples in different cropping practices. (A) Bacteria, (B) Fungi. [file 12866_2022_2638_MOESM2_ESM.tif]
